# 2-(4*H*-1,2,4-Triazol-4-yl)phenol

**DOI:** 10.1107/S1600536810031739

**Published:** 2010-08-18

**Authors:** Wang Zhao, Wei-Wei Zhou, Ming-Jun Song

**Affiliations:** a Department of Physics, Huainan Normal University, Huainan, Anhui 232001, People’s Republic of China; b Department of Chemistry & Chemical Engineering, Huainan Normal University, Huainan, Anhui 232001, People’s Republic of China; c College of Chemical Engineering, Weifang University, Weifang, Shandong 261061, People’s Republic of China

## Abstract

In the title compound, C_8_H_7_N_3_O, the dihedral angle between the benzene and triazole rings is 41.74 (12)°.

## Related literature

For the use of substituted 1,2,4-triazoles as ligands, see: Ouellette *et al.* (2006 ▶); Zhang *et al.* (2005 ▶); Zhou *et al.* (2007 ▶, 2008 ▶). For related structures, see: Wiley & Hart (1953 ▶); Bartlett & Humphrey (1967 ▶); Li *et al.* (2004 ▶); Zhu *et al.* (2000 ▶); Xu *et al.* (2004 ▶).
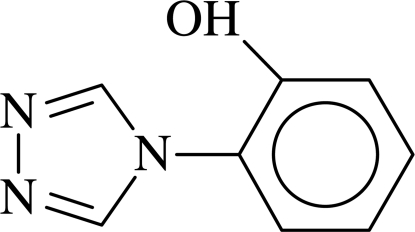


## Experimental

### 

#### Crystal data

C_8_H_7_N_3_O
*M* *_r_* = 161.17
Monoclinic, 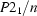

*a* = 7.273 (3) Å
*b* = 14.265 (4) Å
*c* = 7.720 (3) Å
β = 90.93 (3)°
*V* = 800.8 (5) Å^3^
*Z* = 4
Mo *K*α radiation
μ = 0.09 mm^−1^
*T* = 293 K
0.42 × 0.37 × 0.35 mm

#### Data collection

Rigaku Mercury CCD diffractometer
Absorption correction: multi-scan (Sphere in *CrystalClear*; Rigaku, 2002 ▶) *T*_min_ = 0.815, *T*_max_ = 1.000
5037 measured reflections
1460 independent reflections
863 reflections with *I* > 2σ(*I*)
*R*_int_ = 0.057

#### Refinement

*R*[*F* ^2^ > 2σ(*F* ^2^)] = 0.067
*wR*(*F* ^2^) = 0.237
*S* = 1.09
1460 reflections
110 parameters
H-atom parameters constrained
Δρ_max_ = 0.44 e Å^−3^
Δρ_min_ = −0.44 e Å^−3^

### 

Data collection: *CrystalClear* (Rigaku, 2002 ▶); cell refinement: *CrystalClear*; data reduction: *CrystalClear*; program(s) used to solve structure: *SHELXTL* (Sheldrick, 2008 ▶); program(s) used to refine structure: *SHELXTL*; molecular graphics: *SHELXTL*; software used to prepare material for publication: *SHELXTL*.

## Supplementary Material

Crystal structure: contains datablocks I, global. DOI: 10.1107/S1600536810031739/jh2195sup1.cif

Structure factors: contains datablocks I. DOI: 10.1107/S1600536810031739/jh2195Isup2.hkl

Additional supplementary materials:  crystallographic information; 3D view; checkCIF report

## Figures and Tables

**Fig. 1.**
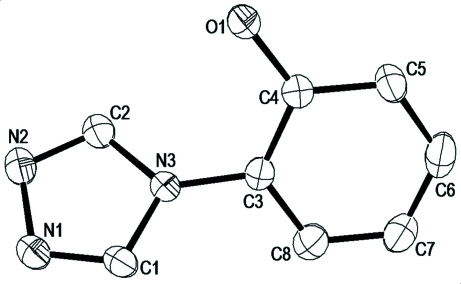
The structure of (I), showing 30% probability displacement ellipsoids and the atom-numbering scheme.

**Fig. 2.**
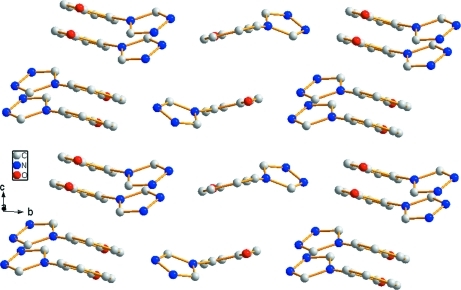
View of the 3-D structure of the title compound.

## supplementary crystallographic information

### Comment

Many compounds with uncommon properties have been widely investigated by using substituted 1,2,4-triazoles ligands, resulting from their rich coordination fashions and broad potential applications in various fields (Ouellette *et al.* (2006); Zhang *et al.* (2005); Zhou *et al.* (2007); Zhou *et al.* (2008)). Substituted 1,2,4-triazoles can be synthesized from different amines and diformylhydrazine. The triazole ring, having strong -donor and weak-acceptor properties, potentially has two different coordination modes through three nitrogen-donor atoms coordinating to metal ions. Recently, we have prepared some new substituted 1,2,4-triazole derivatives and their transition-metal complexes, and we report here the crystal structure analysis of 2-(1*H*-1,2,4-Triazol-4-yl)phenol, (I).

### Experimental

The title compound was prepared by reacting diformylhydrazine (0.6 mmol, 0.053 g) and *o*-aminophenol (0.6 mmol, 0.065 g) in a Telon-lined stainless steel autoclave in a furnace at 443 K for 2 d. The reaction vessel was then cooled to 293 K. The product was isolated and washed with hot water and hot ethanol and black crystals suitable for X-ray diffraction studies were obtained. The crystals are air-stable. Yield based on *o*-aminophenol: 0.062 g, 64%. Elemental analysis (%) for C8H7N3O, found (calculated): C 59.70 (59.61), H 4.25 (4.38), N 26.06 (26.08).

### Refinement

Hydrogen atoms were allowed to ride on their respective parent atoms with C—H distances of 0.93 Å, and were included in the refinement with isotropic displacement parameters *U*_iso_(H) = 1.2*U*_eq_(C).

### Crystal data

**Table:**

C_8_H_7_N_3_O	*F*(000) = 336
*M**_r_* = 161.17	*D*_x_ = 1.337 Mg m^−^^3^
Monoclinic, *P*2_1_/*n*	Mo *K*α radiation, λ = 0.71073 Å
*a* = 7.273 (3) Å	Cell parameters from 1048 reflections
*b* = 14.265 (4) Å	θ = 2.6–27.4°
*c* = 7.720 (3) Å	µ = 0.09 mm^−^^1^
β = 90.93 (3)°	*T* = 293 K
*V* = 800.8 (5) Å^3^	Block, black
*Z* = 4	0.42 × 0.37 × 0.35 mm

### Data collection

**Table:**

Rigaku Mercury CCD diffractometer	1460 independent reflections
Radiation source: rotating-anode generator	863 reflections with *I* > 2σ(*I*)
graphite	*R*_int_ = 0.057
ω scans	θ_max_ = 25.4°, θ_min_ = 3.0°
Absorption correction: multi-scan (Sphere in *CrystalClear*; Rigaku, 2002)	*h* = −8→8
*T*_min_ = 0.815, *T*_max_ = 1.000	*k* = −16→17
5037 measured reflections	*l* = −9→8

### Refinement

**Table:**

Refinement on *F*^2^	Secondary atom site location: difference Fourier map
Least-squares matrix: full	Hydrogen site location: inferred from neighbouring sites
*R*[*F*^2^ > 2σ(*F*^2^)] = 0.067	H-atom parameters constrained
*wR*(*F*^2^) = 0.237	*w* = 1/[σ^2^(*F*_o_^2^) + (0.144*P*)^2^] where *P* = (*F*_o_^2^ + 2*F*_c_^2^)/3
*S* = 1.09	(Δ/σ)_max_ < 0.001
1460 reflections	Δρ_max_ = 0.44 e Å^−^^3^
110 parameters	Δρ_min_ = −0.44 e Å^−^^3^
0 restraints	Extinction correction: *SHELXTL* (Sheldrick, 2008), Fc^*^=kFc[1+0.001xFc^2^λ^3^/sin(2θ)]^-1/4^
Primary atom site location: structure-invariant direct methods	Extinction coefficient: 0.08 (3)

### Special details

**Table:**

Geometry. All e.s.d.'s (except the e.s.d. in the dihedral angle between two l.s. planes) are estimated using the full covariance matrix. The cell e.s.d.'s are taken into account individually in the estimation of e.s.d.'s in distances, angles and torsion angles; correlations between e.s.d.'s in cell parameters are only used when they are defined by crystal symmetry. An approximate (isotropic) treatment of cell e.s.d.'s is used for estimating e.s.d.'s involving l.s. planes.
Refinement. Refinement of *F*^2^ against ALL reflections. The weighted *R*-factor *wR* and goodness of fit *S* are based on *F*^2^, conventional *R*-factors *R* are based on *F*, with *F* set to zero for negative *F*^2^. The threshold expression of *F*^2^ > σ(*F*^2^) is used only for calculating *R*-factors(gt) *etc*. and is not relevant to the choice of reflections for refinement. *R*-factors based on *F*^2^ are statistically about twice as large as those based on *F*, and *R*- factors based on ALL data will be even larger.

### Fractional atomic coordinates and isotropic or equivalent isotropic displacement parameters (Å^2^)

**Table:**

	*x*	*y*	*z*	*U*_iso_*/*U*_eq_	
C1	0.5881 (5)	0.7175 (2)	0.7593 (5)	0.0559 (10)	
H1A	0.5053	0.7418	0.8381	0.067*	
C2	0.7294 (5)	0.6248 (2)	0.5912 (4)	0.0510 (10)	
H2A	0.7633	0.5718	0.5293	0.061*	
C3	0.4535 (4)	0.5571 (2)	0.7425 (4)	0.0423 (8)	
C4	0.5137 (4)	0.4665 (2)	0.7748 (4)	0.0448 (9)	
C5	0.3858 (5)	0.4004 (2)	0.8286 (4)	0.0542 (10)	
H5A	0.4233	0.3394	0.8527	0.065*	
C6	0.2034 (5)	0.4252 (3)	0.8463 (5)	0.0651 (11)	
H6A	0.1194	0.3807	0.8837	0.078*	
C7	0.1443 (5)	0.5148 (3)	0.8091 (5)	0.0633 (11)	
H7A	0.0210	0.5308	0.8200	0.076*	
C8	0.2698 (5)	0.5804 (3)	0.7557 (5)	0.0582 (10)	
H8A	0.2309	0.6408	0.7284	0.070*	
N1	0.7219 (4)	0.76432 (19)	0.6942 (4)	0.0632 (10)	
N2	0.8157 (4)	0.70426 (19)	0.5852 (4)	0.0618 (10)	
N3	0.5836 (3)	0.62837 (17)	0.6985 (3)	0.0443 (8)	
O1	0.6918 (3)	0.44556 (15)	0.7550 (3)	0.0582 (8)	
H1B	0.7472	0.4923	0.7229	0.087*	

### Atomic displacement parameters (Å^2^)

**Table:**

	*U*^11^	*U*^22^	*U*^33^	*U*^12^	*U*^13^	*U*^23^
C1	0.068 (2)	0.0332 (18)	0.068 (2)	0.0041 (15)	0.0238 (18)	0.0022 (16)
C2	0.057 (2)	0.0414 (18)	0.0553 (19)	−0.0010 (15)	0.0129 (17)	0.0025 (15)
C3	0.0378 (18)	0.0426 (17)	0.0466 (17)	−0.0030 (14)	0.0046 (13)	0.0028 (14)
C4	0.0428 (19)	0.0414 (19)	0.0504 (17)	−0.0018 (14)	0.0042 (14)	0.0001 (14)
C5	0.060 (2)	0.0429 (19)	0.060 (2)	−0.0089 (15)	0.0074 (18)	0.0071 (16)
C6	0.058 (2)	0.075 (3)	0.063 (2)	−0.024 (2)	0.0154 (18)	−0.005 (2)
C7	0.047 (2)	0.070 (3)	0.072 (2)	−0.0036 (18)	0.0121 (18)	−0.001 (2)
C8	0.050 (2)	0.058 (2)	0.067 (2)	0.0034 (16)	0.0065 (17)	0.0065 (17)
N1	0.073 (2)	0.0368 (16)	0.081 (2)	−0.0025 (14)	0.0284 (17)	−0.0018 (15)
N2	0.065 (2)	0.0458 (17)	0.076 (2)	−0.0100 (14)	0.0222 (16)	−0.0004 (14)
N3	0.0449 (16)	0.0329 (15)	0.0553 (16)	0.0000 (11)	0.0117 (12)	0.0012 (11)
O1	0.0451 (15)	0.0400 (13)	0.0897 (19)	0.0004 (10)	0.0081 (12)	0.0087 (12)

### Geometric parameters (Å, °)

**Table:**

C1—N1	1.289 (4)	C4—C5	1.393 (4)
C1—N3	1.356 (4)	C5—C6	1.382 (5)
C1—H1A	0.9300	C5—H5A	0.9300
C2—N2	1.297 (4)	C6—C7	1.378 (6)
C2—N3	1.357 (4)	C6—H6A	0.9300
C2—H2A	0.9300	C7—C8	1.375 (5)
C3—C8	1.382 (4)	C7—H7A	0.9300
C3—C4	1.385 (5)	C8—H8A	0.9300
C3—N3	1.433 (4)	N1—N2	1.388 (4)
C4—O1	1.340 (4)	O1—H1B	0.8200
			
N1—C1—N3	111.4 (3)	C7—C6—C5	120.9 (3)
N1—C1—H1A	124.3	C7—C6—H6A	119.5
N3—C1—H1A	124.3	C5—C6—H6A	119.5
N2—C2—N3	111.9 (3)	C8—C7—C6	119.2 (3)
N2—C2—H2A	124.1	C8—C7—H7A	120.4
N3—C2—H2A	124.1	C6—C7—H7A	120.4
C8—C3—C4	120.9 (3)	C7—C8—C3	120.4 (4)
C8—C3—N3	119.3 (3)	C7—C8—H8A	119.8
C4—C3—N3	119.8 (3)	C3—C8—H8A	119.8
O1—C4—C3	119.4 (3)	C1—N1—N2	107.4 (3)
O1—C4—C5	122.3 (3)	C2—N2—N1	105.9 (3)
C3—C4—C5	118.4 (3)	C1—N3—C2	103.4 (2)
C6—C5—C4	120.2 (3)	C1—N3—C3	126.6 (3)
C6—C5—H5A	119.9	C2—N3—C3	130.0 (3)
C4—C5—H5A	119.9	C4—O1—H1B	109.5
